# Teste calprotectina fecal semiquantitativa na síndrome do intestino irritável pós-infecciosa e não pós-infecciosa: estudo transversal: Semiquantitative fecal calprotectin test in postinfectious and non-postinfectious irritable bowel syndrome: cross-sectional study

**DOI:** 10.1590/1516-3180.2014.8000815

**Published:** 2014-12-19

**Authors:** Liliana-Elisabeta David, Teodora Surdea-Blaga, Dan-Lucian Dumitrascu

**Affiliations:** I BSc. Doctoral Student and Head Nurse, Second Department of Internal Medicine, “Iuliu Hatieganu” University of Medicine and Pharmacy, Cluj-Napoca, Romania.; II MD, MSc. Doctoral Student and Attending Physician, Second Department of Internal Medicine, “Iuliu Hatieganu” University of Medicine and Pharmacy, Cluj-Napoca, Romania.; III MD, PhD. Head of Department, Second Department of Internal Medicine, “Iuliu Hatieganu” University of Medicine and Pharmacy, Cluj-Napoca, Romania.

**Keywords:** Leukocyte L1 antigen complex, Inflammation, Inflammatory bowel diseases, Irritable bowel syndrome, Gastroenteritis, Complexo antígeno L1 leucocitário, Inflamação, Doenças inflamatórias intestinais, Síndrome do intestino irritável, Gastroenterite

## Abstract

**CONTEXT AND OBJECTIVE:**

The presence of a certain degree of inflammation in the gut wall is now accepted in irritable bowel syndrome (IBS). Fecal calprotectin is considered to be a reliable test for detecting intestinal inflammation. Our aim was to assess the presence of inflammation in postinfectious IBS (PI-IBS), compared with non-postinfectious IBS (NPI-IBS). A secondary objective was to determine the usefulness of a rapid fecal calprotectin test in inflammatory bowel diseases (IBD).

**DESIGN AND SETTING:**

This was a cross-sectional study. Patients with IBS and IBD at a single tertiary gastroenterology center were prospectively included in this study.

**METHODS:**

116 patients with Rome III IBS score (76 females; 48 ± 12 years) were investigated; 24 patients (15 females) had PI-IBS. Intestinal inflammation was assessed using the semiquantitative fecal calprotectin test. The results were expressed as T1, T2 or T3 according to the severity of inflammation (< 15 µg/g; 15-60 µg/g; > 60 µg/g). Using the same test, we evaluated 20 patients with IBD (12 males; 47 ± 13 years).

**RESULTS:**

None of the patients with IBS had a T2 or T3 positive test. Among PI-IBS patients, 33% had a T1 positive test. Among NPI-IBS patients, 9.8% had a T1 positive test, which was significantly different to PI-IBS. The calprotectin test was positive in all IBD patients: 80% with T3, 10% with T2 and 10% with T1.

**CONCLUSIONS:**

Using a semiquantitative test for fecal calprotectin, positive tests were more frequent in PI-IBS patients than in NPI-IBS patients.

## RESUMO

**CONTEXTO E OBJETIVO:**

A presença de certo grau de inflamação na parede do intestino é agora aceita na síndrome do intestino irritável (SII). A calprotectina fecal é considerada teste confiável para detectar inflamação intestinal. Nosso objetivo foi avaliar a presença de inflamação na SII pós-infecciosa (SII-PI), em comparação com a SII não pós-infecciosa (SII-NPI). Um objetivo secundário foi determinar a utilidade de um teste rápido fecal da calprotectina em doenças inflamatórias intestinais (DII).

**TIPO DE ESTUDO E LOCAL:**

Este foi um estudo transversal. Pacientes com SII e DII em um único centro terciário de gastroenterologia foram prospectivamente incluídos neste estudo.

**MÉTODOS:**

116 pacientes com escore Roma III de SII (76 mulheres, 48 ± 12 anos) foram investigados; 24 pacientes (15 mulheres) tinham SII-PI. Inflamação intestinal foi avaliada pelo teste semi-quantitativo de calprotectina fecal. Os resultados foram expressos como T1, T2 ou T3 de acordo com a gravidade da inflamação (< 15 µg/g; 15-60 mg/g; > 60 mg/g). Usando o mesmo teste, foram avaliados 20 pacientes com DII (12 homens, 47 ± 13 anos).

**RESULTADOS:**

Nenhum dos pacientes com SII teve um teste positivo T2 ou T3. Na PI-IBS, 33% tiveram um teste positivo T1. Entre os pacientes SII-NPI, teste T1 positivo estava presente em 9,8%, taxa significativamente diferente quando comparada com SII-PI. O teste de calprotectina foi positivo em todos os pacientes com DII: 80% com T3, 10% com T2 e 10% com T1.

**CONCLUSÕES:**

Usando teste semi-quantitativo para calprotectina fecal, relatamos positividade em pacientes SII-PI com mais frequência que em pacientes SII-NPI.

## INTRODUCTION

It is now accepted that a certain degree of inflammation in the gut wall is present in irritable bowel syndrome.^1^ Since irritable bowel syndrome is a functional disorder, with a very good prognosis in terms of survival, it is preferable to use noninvasive tests in order to evaluate the presence of inflammation. More than a decade ago, measurement of calprotectin in feces was proposed as a surrogate marker of intestinal inflammation.^2^ Calprotectin represents 60% of granulocytic cytosolic proteins, and therefore its concentration reflects the neutrophil migration in the gastrointestinal tract. Increased levels of calprotectin indicate intestinal inflammation, but it is not disease-specific.^3^

Over the last few years, several studies have focused on evaluating the value of fecal calprotectin for detecting mucosal inflammation, especially in patients with inflammatory bowel disease, both in an active phase and in clinical remission. Patients with clinically quiescent inflammatory bowel disease have some degree of mucosal inflammation,^4^ as proved by high levels of fecal calprotectin. For example, Sipponen et al. reported that there were high levels of calprotectin (up to 1000 mcg/g) in 13% of their inflammatory bowel disease patients who were in clinical remission.^5^ Fecal calprotectin also has prognostic value, such that the probability of remaining in clinical remission is higher when the fecal calprotectin level is low.^5^ There is also evidence that patients with mucosal healing seen through endoscopy have lower or normal fecal calprotectin levels.^6^^,^^7^ Fecal calprotectin levels also become increased in other organic disorders such as small bowel enteropathy, microscopic colitis, infectious diarrhea, segmental colitis associated with diverticulosis and colorectal cancer.^8^

Fecal calprotectin levels are low both in irritable bowel syndrome patients and in healthy controls.^8^^,^^9^ Several studies have shown that increased fecal calprotectin levels can differentiate between organic colonic diseases and nonorganic disease (especially irritable bowel syndrome), in symptomatic patients.^8^^,^^10^ Using a cutoff of 10 mg/l, fecal calprotectin had sensitivity of 89% and specificity of 79% for organic disease.^8^

Postinfectious irritable bowel syndrome is a specific type of irritable bowel syndrome, acknowledged by the Rome working committees.^1^ However, there is evidence that postinfectious irritable bowel syndrome patients differ from non-postinfectious irritable bowel syndrome patients by having a low level of intestinal inflammation.^11^ Persistent inflammation after the acute infection may be important in the pathogenesis of postinfectious irritable bowel syndrome.

We started from the hypothesis that fecal calprotectin would be positive in a higher proportion of patients with postinfectious irritable bowel syndrome, compared with non-postinfectious irritable bowel syndrome patients.

## OBJECTIVE

The main objective of our study was to find out whether there were any differences in fecal calprotectin levels in patients with postinfectious irritable bowel syndrome, compared with non-postinfectious irritable bowel syndrome patients, using a rapid semiquantitative fecal calprotectin test. A second objective of the study was to investigate the usefulness of a rapid and inexpensive fecal calprotectin test in a group of patients with obvious intestinal inflammation, such as patients with inflammatory bowel diseases.

## METHODS

### Study design and setting

This was a cross-sectional study carried out in a public hospital for six months in 2012.

### Subjects

#### Irritable bowel syndrome patients

A total of 116 consecutive irritable bowel syndrome patients referred to a single tertiary gastroenterology department were prospectively included in this study after they gave their informed consent. The patients were diagnosed as having irritable bowel syndrome in accordance with the Rome III criteria, i.e. abdominal pain present on at least three days per month on average, over the last three months, with the onset of symptoms at least six months earlier, in the absence of any structural or biochemical cause.^1^ Among the 116 patients with irritable bowel syndrome, 76 (65.5%) were females, and the mean age was 48 ± 12 years. To our knowledge, there are no data in the literature that have compared the levels of fecal calprotectin in postinfectious irritable bowel syndrome and non-postinfectious irritable bowel syndrome patients. This was a pilot study and we used a convenience sample.

The patients with irritable bowel syndrome were grouped into postinfectious irritable bowel syndrome and non-postinfectious irritable bowel syndrome. The diagnosis of postinfectious irritable bowel syndrome was established by asking the patients about their medical history over the year before the onset of irritable bowel syndrome.^12^ Patients were assigned to the postinfectious irritable bowel syndrome group if they recognized a triggering event consisting of an acute episode of gastroenteritis (nausea, vomiting and diarrhea), during the year before the irritable bowel syndrome symptoms developed. They were assigned to the non-postinfectious irritable bowel syndrome group, if they did not recall such an episode in the past. Twenty-four patients (20.6%) presented postinfectious irritable bowel syndrome, of whom 15 were female. There were no differences regarding age and gender between the postinfectious irritable bowel syndrome group and the non-postinfectious irritable bowel syndrome group.

Patients with coexisting cardiovascular, pulmonary, hepatic, renal or musculoskeletal disease, severe immune deficiency, malignancy or alcohol abuse, or who were receiving non-steroidal anti-inflammatory drugs, were excluded from the study because these conditions may be associated with intestinal inflammation.^13^^,^^14^ Patients with menstrual or nasal bleeding during the five days prior to fecal testing were also excluded, since blood in the feces could increase the calprotectin levels.^15^^,^^16^

#### Inflammatory bowel disease patients

Fecal calprotectin levels are high in patients with inflammatory bowel diseases,^5^ and vary with the severity of inflammation. Fecal calprotectin is used in current practice to monitor the evolution of patients with inflammatory bowel diseases, but the costs of ELISA (enzyme-linked immunosorbent assay) tests are quite high. In order to achieve the second objective of this study and to confirm previously published data,^3^^,^^5^ we applied a rapid and inexpensive fecal calprotectin test to a small group of patients with inflammatory bowel diseases.

We selected 20 consecutive patients (eight females) with inflammatory bowel diseases who had been admitted to our department: 15 with ulcerative colitis, 3 with Crohn’s disease and 2 with unspecified colitis. The mean age of the inflammatory bowel disease group was 47 ± 13 years. The diagnosis of inflammatory bowel disease was based on the macroscopic appearance of the colonic mucosa during lower gastrointestinal endoscopy, and was confirmed from histopathological analysis on colonic biopsies. The patients were seen either at the first flare of colitis, or during a long remission without treatment. Patients with Crohn’s disease complications (fistulae, abscesses or symptomatic intestinal strictures requiring surgery) were excluded.

#### Assessment of fecal calprotectin

We assessed intestinal inflammation by means of a rapid, semiquantitative commercially available test (CalDetect, SOFAR, Trezzano Rosa, Italy), which uses an immunochromatographic method to detect the presence of calprotectin in the feces. There are studies showing that rapid tests are useful as a screening test for excluding gastrointestinal inflammation when the cutoff of 15 mcg/g is used, with a negative predictive value of 94%.^17^ The test can be performed on the same day, or within a maximum of one week if the feces are kept at between 2 and 8 °C. Its major advantage is that the result is available after 15 minutes. We always used the test within the first hour after obtaining the sample of feces. The result can be negative or positive.

According to the producer’s specifications, the presence of one red control band (C) alone indicates that calprotectin is not present in the feces, and we referred to this situation as a clearly negative test. The presence of two color bands (C and T1) corresponds to fecal calprotectin < 15 mcg/g, and was referred to in our study as a T1 positive test. The presence of three color bands (C, T1 and T2) indicates fecal calprotectin of between 15 and 60 mcg/g and was referred to in our study as a T2 positive test. The presence of four color bands (C, T1, T2 and T3) indicates fecal calprotectin > 60 mcg/g, and was referred to in our study as a T3 positive test.^18^

**Figure 1.**
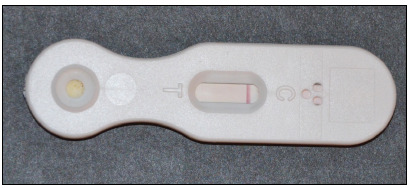
Plaque showing a negative fecal calprotectin test. Only the control band is visible.

**Figure 2.**
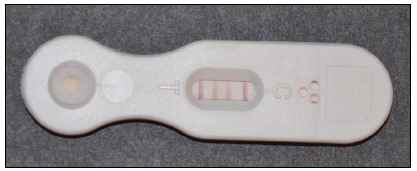
Plaque showing a T3 positive calprotectin test. Four color bands are visible, C corresponds to the control band (C).

Thus, according to this test, the fecal calprotectin assessment may give one of the following results: negative or positive. If positive, the test returns one of three values: T1 if fecal calprotectin < 15 mcg/g (suggesting minimal inflammation); T2 if fecal calprotectin = 15-60 mcg/g (moderate inflammation); T3 if fecal calprotectin > 60 mcg/g (severe inflammation)^18^ (Figures 1 and 2).

All patients, both those with irritable bowel syndrome and those with inflammatory bowel disease, were evaluated using this fecal calprotectin test, but no comparison between the two groups was made.

### Statistical analysis

Descriptive statistics (mean ± standard deviation) were used for parametric data. To compare the proportions of positive tests in cases of postinfectious irritable bowel syndrome versus cases of non-postinfectious irritable bowel syndrome, we used the Z test for proportions. An error probability of P < 0.05 was considered statistically significant.

### Ethical issues

This study was conducted in accordance with the Declaration of Helsinki regarding human studies. All patients gave their informed consent and the study received approval from our institution’s Ethics Committee.

## RESULTS

### Fecal calprotectin in irritable bowel syndrome patients

**Figure 3.**
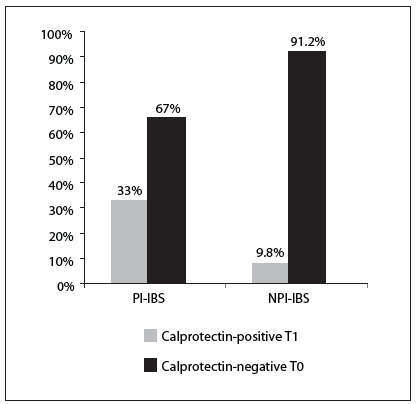
Proportions of patients with a mild T1 positive fecal calprotectin test and a T0 negative fecal calprotectin test in cases of postinfectious irritable bowel syndrome (PI-IBS) and non-postinfectious irritable bowel syndrome (NPI-IBS).

Eight patients (33%) out of 24 with postinfectious irritable bowel syndrome and nine patients (9.8%) out of 92 with non-postinfectious irritable bowel syndrome had a T1 positive calprotectin test (Figure 3). None of the patients had a T2 or a T3 positive fecal calprotectin test, and therefore none of the irritable bowel syndrome patients had moderate or severe inflammation. All the other patients with irritable bowel syndrome, i.e. both with postinfectious and non-postinfectious irritable bowel syndrome, had a negative fecal calprotectin test (T0). The proportion of patients with a mild positive fecal calprotectin test (T1) was significantly higher (Z = 2.9; P < 0.001) in the postinfectious irritable bowel syndrome group than in the non-postinfectious irritable bowel syndrome group (Figure 3).

The post-hoc power analysis on this test indicated a power of 0.65.

### Fecal calprotectin in inflammatory bowel disease patients

**Figure 4.**
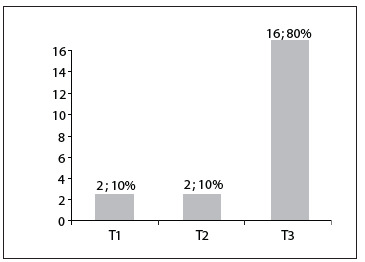
Results from fecal calprotectin testing among patients with irritable bowel diseases. T1, mild inflammation; T2, moderate inflammation; and T3, severe inflammation. The results are expressed as the number of patients; and the rate from 20 patients with inflammatory bowel diseases.

Out of the 20 patients with inflammatory bowel disease, none had a negative fecal calprotectin test. The majority of the inflammatory bowel disease patients (16/20) had a T3 positive fecal calprotectin test, thus suggesting severe inflammation. The results are summarized in Figure 4.

## DISCUSSION

The usefulness of calprotectin for detecting intestinal inflammation is accepted worldwide. Our study performed both on irritable bowel syndrome patients and on inflammatory bowel disease patients showed that even a simpler method such as a semiquantitative test for fecal calprotectin is useful for detecting intestinal inflammation. In addition, using this semiquantitative method, our study is one of the first to demonstrate that patients with postinfectious irritable bowel syndrome have a higher degree of inflammation than do patients with non-postinfectious irritable bowel syndrome. A limited number of studies have used this immunochromatographic method for detecting colonic inflammation.^3^^,^^19^ The semiquantitative test had sensitivity and specificity comparable to ELISA-based fecal calprotectin tests for detecting inflammation in patients with ulcerative colitis and Crohn’s disease.^3^

In our study, 20% of the patients were classified as presenting postinfectious irritable bowel syndrome. Our result was similar to other observations regarding the incidence of postinfectious irritable bowel syndrome following infectious gastroenteritis (in the 12^th^ month after the acute episode), which have ranged from 13% to 31%.^12^^,^^20^ In our irritable bowel syndrome group, 14.6% of the patients had a positive fecal calprotectin test (T1 positive), thus suggesting mild inflammation. Half of them had postinfectious irritable bowel syndrome. Another study that used this semiquantitative method among irritable bowel syndrome patients reported that there was no increase in fecal calprotectin levels in irritable bowel syndrome in relation to healthy controls.^21^ Our study showed that a higher proportion of patients with postinfectious irritable bowel syndrome had a positive calprotectin test, compared with patients with non-postinfectious irritable bowel syndrome. This suggests that after an acute episode of enterocolitis, there is a degree of persistent inflammation.

Other studies have also reported mild inflammation in cases of irritable bowel syndrome. Tibble et al. evaluated fecal calprotectin (using the ELISA method for quantification) in 602 patients with gastrointestinal complaints, of whom 339 were classified as presenting nonorganic disease (all of them had irritable bowel syndrome symptoms), and 263 as presenting organic disease. Fecal calprotectin < 10 mg/l was considered normal. In the nonorganic group, the median fecal calprotectin level was 4 mg/l, with a range of 1-50 mg/l.^8^ Thus, several irritable bowel syndrome patients had fecal calprotectin > 10 mg/l, but < 50 mg/l, which suggested that there was some degree of inflammation in these irritable bowel syndrome cases. These authors did not provide further information regarding the postinfectious status of these patients.^8^ Similar results were reported in another study. The range of fecal calprotectin detected using ELISA was 0-24 mcg/g, with a median value of 6 mcg/g. The highest sensitivity and specificity of fecal calprotectin for differentiating organic from functional disorders was observed using a cutoff value of 24.3 mcg⁄g.^22^

In our study, we used a semiquantitative test to detect fecal calprotectin, with positive tests expressed in mcg/g. Thus, we cannot directly compare our results with those obtained using the ELISA method (values expressed in mg/l). However, we can say that, in the same way as shown in Tibble et al. study,^8^ fecal calprotectin was positive in some of our irritable bowel syndrome patients, although only low levels were detected.

Both in the postinfectious irritable bowel syndrome and in the non-postinfectious irritable bowel syndrome, there is evidence of some degree of inflammation. Some studies have reported increased numbers of intraepithelial T lymphocytes and enterochromaffin cells in rectal biopsy specimens, 12 weeks after acute gastroenteritis, compared with the cell counts in control subjects.^23^^,^^24^^,^^25^ In another study, the number of enterochromaffin cells was significantly higher in a group with postinfectious irritable bowel syndrome than in a group with non-postinfectious irritable bowel syndrome, but the number of T lymphocytes was similar.^26^ Our study showed that a higher proportion of patients with postinfectious irritable bowel syndrome had a positive calprotectin test, compared with patients with non-postinfectious irritable bowel syndrome (33% versus 10%). This suggests that the degree of intestinal inflammation is higher in patients with postinfectious irritable bowel syndrome than in those with non-postinfectious irritable bowel syndrome.

The usefulness of fecal calprotectin testing in cases of inflammatory bowel disease has already been proven. We also tested this semiquantitative method on inflammatory bowel disease patients. Out of 20 patients with inflammatory bowel disease, 80% had a T3 positive test, i.e. fecal calprotectin > 60 mcg/g. Using ELISA testing on fecal calprotectin, a high cutoff value set at 100 mcg/g had better accuracy than the usual cutoff limit of 50 mcg/g.^27^ Our cutoff value for moderate to severe inflammation (as seen in inflammatory bowel disease) was 60 mcg/g. Only 20% of our inflammatory bowel disease patients had fecal calprotectin < 60 mcg/g. Our results from inflammatory bowel disease patients were similar to those reported in other studies, in which the calprotectin levels were rarely below 60 mcg/g, and varied widely between 54 and 6032 mcg/g.^28^ A large study (including 823 patients) that used CalDetect, reported that only 7% of patients with active Crohn’s disease had a calprotectin level < 15 ng/ml, while among patients with inactive Crohn’s disease, 89% had a calprotectin level < 15 ng/ml. These results were very close to those observed using ELISA methods, thus resulting in very good performance for this rapid test.^3^

In cases of irritable bowel syndrome, many patients are still concerned with abdominal pain or diarrhea, and some of them undergo repeated colonoscopies. Patients with inflammatory bowel disease often need repeated colonoscopies for follow-up and evaluation of treatment efficacy. It is obvious that in these cases, fecal calprotectin testing is preferable, since it is inexpensive, noninvasive, rapid, sensitive and specific for intestinal inflammation.

On the other hand, fecal calprotectin testing has some disadvantages. False positive results may occur. Thus, use of non-steroidal anti-inflammatory drugs can increase fecal calprotectin levels.^29^^,^^30^ Age is also thought to influence calprotectin levels.^15^ The presence of blood in stools (at least 100 ml/day) increases fecal calprotectin levels, and therefore testing should be avoided in patients with menstrual or nasal bleeding.^15^^,^^16^ It is worth mentioning that several authors have showed that there is considerable variability of calprotectin levels in the same fecal sample, or in different samples on consecutive days, from the same patients.^31^ This disadvantage can be overcome by repeating this inexpensive and rapid test several times.

This study shows the usefulness of a simple and inexpensive semiquantitative fecal test for assessing the degree of intestinal inflammation in different pathological bowel diseases. It is consistent with previous data that showed that in irritable bowel syndrome, inflammation may be present to a lower degree.^32^ We showed that postinfectious irritable bowel syndrome more frequently presented positive fecal calprotectin than did non-postinfectious irritable bowel syndrome, i.e. in cases of postinfectious irritable bowel syndrome, intestinal inflammation may be more frequently encountered than in cases of non-postinfectious irritable bowel syndrome.

Our study has several limitations. Since there are no other data in the literature regarding use of this rapid test among patients with irritable bowel syndrome, we used a convenience sample. For objectivity, we determined the post-hoc power of the test, which was quite low, even if the differences observed between the postinfectious and non-postinfectious irritable bowel syndrome groups were statistically significant. We are aware that it would have been ideal to have had higher power and, implicitly, a larger sample size. This pilot study shows that in order to obtain a power greater than 0.8, larger sample populations of irritable bowel syndrome patients are needed. In addition, we did not compare the results from the rapid fecal calprotectin test with a more precise determination of fecal calprotectin, such as an ELISA quantitative test. However, patients with irritable bowel syndrome had fecal calprotectin < 15 mcg/g, similar to data reported in the literature. One advantage of the test that we used was that the cutoff values could differentiate between low levels of fecal calprotectin. Our results confirmed previously reported data regarding fecal calprotectin in inflammatory bowel disease, and showed that this rapid and inexpensive test can be used even among these patients.

The semiquantitative test for fecal calprotectin is useful and affordable within general practice and therefore it can be used to differentiate functional from organic diseases. Among irritable bowel syndrome patients, calprotectin is more frequently present in cases of postinfectious irritable bowel syndrome than in cases of non-postinfectious irritable bowel syndrome. Future directions evolving from this study will involve using rapid qualitative tests to investigate the differences in fecal calprotectin levels between postinfectious irritable bowel syndrome and non-postinfectious irritable bowel syndrome. Whether identifying patients with irritable bowel syndrome with mild inflammation would change their management remains to be determined.

## CONCLUSIONS

A simple, rapid and inexpensive test such as the semiquantitative fecal calprotectin assay is able to differentiate non-postinfectious irritable bowel syndrome, with absence or low levels of inflammation, from postinfectious irritable bowel syndrome patients, whose calprotectin levels are higher. The test can also be used for identifying patients with severe intestinal inflammation, such as patients with inflammatory bowel diseases.
